# Supplementary material for: Genome-wide screening identified SEC61A1 as an essential factor for mycolactone-dependent apoptosis in human premonocytic THP-1 cells
Source: PLoS Negl Trop Dis. 2022 Aug 8;16(8):e0010672. doi: 10.1371/journal.pntd.0010672 (PMC9387930; doi:10.1371/journal.pntd.0010672)

**S1 Fig. Ranking of the candidate genes identified by GeCKO screening.** Genes were ranked according to the p-value of MAGeCK screening. Lower RRA p-values indicate a stronger positive selection of the corresponding gene. The top 10 ranked genes are labeled with red dots. Genes with a p <0.05 are shown in the gray area. Source data are provided in S1 Data.


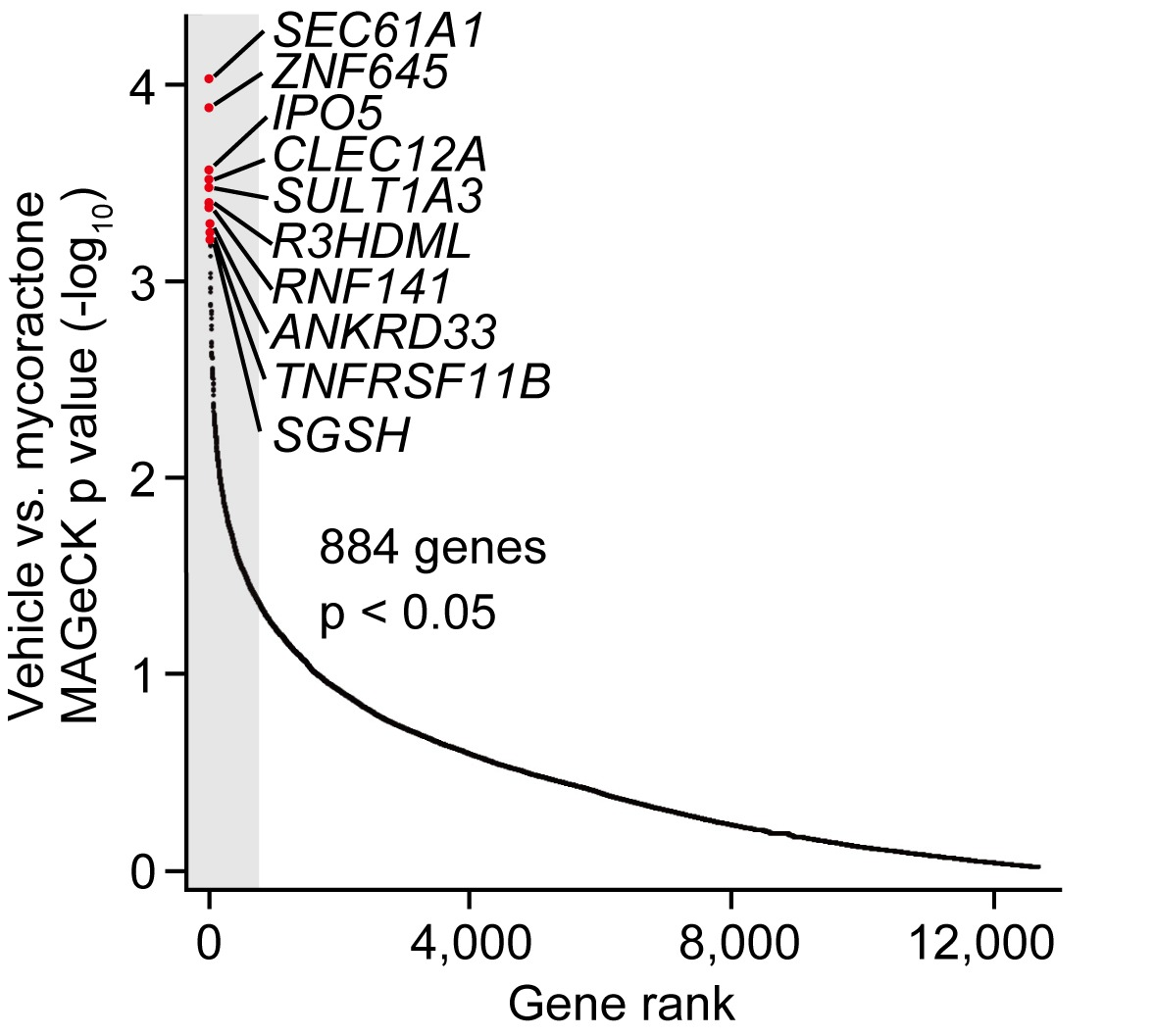

Supplement: S1 Fig — Genes were ranked according to the p-value of MAGeCK screening. Lower RRA p-values indicate a stronger positive selection of the corresponding gene. The top 10 ranked genes are labeled with red dots. Genes with a p <0.05 are shown in the gray area. Source data are provided in S1 Data. (DOCX) [file pntd.0010672.s001.docx]
