# Supplementary material for: Genome-wide screening identified SEC61A1 as an essential factor for mycolactone-dependent apoptosis in human premonocytic THP-1 cells
Source: PLoS Negl Trop Dis. 2022 Aug 8;16(8):e0010672. doi: 10.1371/journal.pntd.0010672 (PMC9387930; doi:10.1371/journal.pntd.0010672)
Supplement: S2 Fig — (A) Cell lysates were prepared from control EGFP-knockout and SEC61A1-knockout THP-1 cells and analyzed by immunoblotting with Abs against SEC61A1. β-Actin was used as a loading control. (B) PCR amplification of genomic DNA from control EGFP-knockout and SEC61A1-knockout THP-1 cells, using a FWD primer targeting the SEC61A1 sgRNA binding site and a BWD primer targeting 100 bp downstream. The higher PCR annealing temperature was set to 65°C for 15 cycles. (C) Sequences of the SEC61A1 sgRNA targeting site from PCR products, which were amplified from genomic DNA of control EGFP-knockout and SEC61A1-knockout THP-1 cells. The sequences are compared to the reference human genome sequence. N means that the nucleotide was not determined by sequencing due to multiple mutations inserted near the PAM sequence (protospacer adjacent motif, which is a CCG DNA sequence). (D) Cell proliferation assay. The cell proliferation assay indicated that control, EGFP-knockout and SEC61A1-knockout THP-1 cell numbers were distinctly increased in a time-dependent manner during the 5-day culture. The cell number and viability were measured by the trypan blue exclusion assay using an automatic cell counter (n = 6). ***: p < 0.005 compared with the cell number of control THP-1 cells. †***: p < 0.005 compared with the cell number of EGFP-knockout THP-1 cells. (DOCX) [file pntd.0010672.s002.docx]

**S2 Fig. *SEC61A1*-deleted THP-1 cells were generated using the CRISPR/Cas9 genome editing system.** (A) Cell lysates were prepared from control *EGFP*-knockout and *SEC61A1*-knockout THP-1 cells and analyzed by immunoblotting with Abs against SEC61A1. β-Actin was used as a loading control. (B) PCR amplification of genomic DNA from control *EGFP*-knockout and *SEC61A1*-knockout THP-1 cells, using a FWD primer targeting the *SEC61A1* sgRNA binding site and a BWD primer targeting 100 bp downstream. The higher PCR annealing temperature was set to 65°C for 15 cycles. (C) Sequences of the *SEC61A1* sgRNA targeting site from PCR products, which were amplified from genomic DNA of control *EGFP*-knockout and *SEC61A1*-knockout THP-1 cells. The sequences are compared to the reference human genome sequence. N means that the nucleotide was not determined by sequencing due to multiple mutations inserted near the PAM sequence (protospacer adjacent motif, which is a CCG DNA sequence). (D) Cell proliferation assay. The cell proliferation assay indicated that control, *EGFP*-knockout and *SEC61A1*-knockout THP-1 cell numbers were distinctly increased in a time-dependent manner during the 5-day culture. The cell number and viability were measured by the trypan blue exclusion assay using an automatic cell counter (n=6). ***: p < 0.005 compared with the cell number of control THP-1 cells. ^†^***: p < 0.005 compared with the cell number of *EGFP*-knockout THP-1 cells.

**
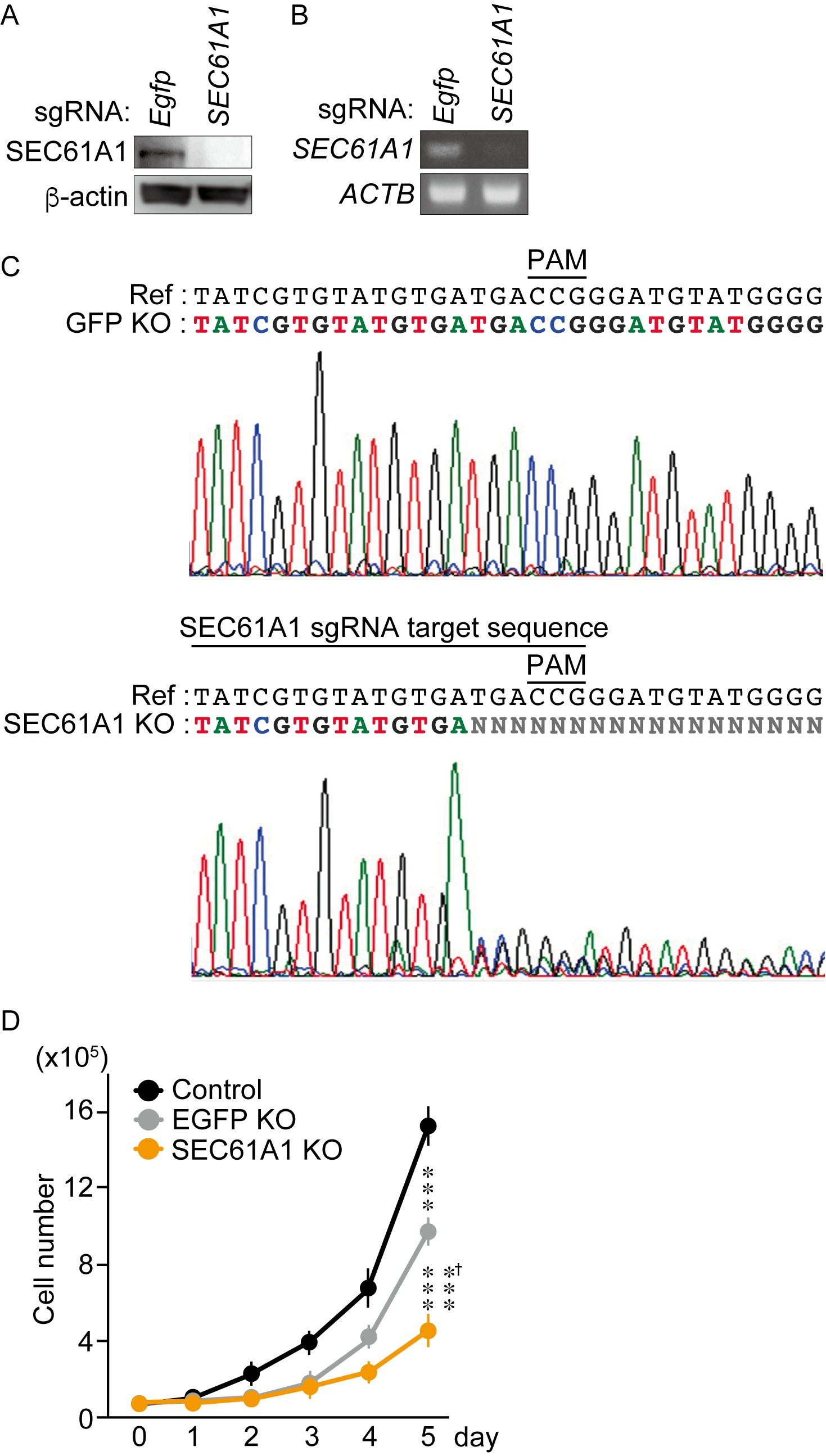
**
