# Supplementary material for: Genome-wide screening identified SEC61A1 as an essential factor for mycolactone-dependent apoptosis in human premonocytic THP-1 cells
Source: PLoS Negl Trop Dis. 2022 Aug 8;16(8):e0010672. doi: 10.1371/journal.pntd.0010672 (PMC9387930; doi:10.1371/journal.pntd.0010672)

**S3 Fig. Mycolactone induced the expression of ER-stress-related genes and proapoptotic genes.** The THP-1 cells were treated with 30 or 300 ng/mL mycolactone for the indicated periods. mRNA levels were analyzed by real-time RT-PCR and normalized to those of *GAPDH*. The expression levels of ER-stress-related genes (*ATF4* and *DDIT3*) and pro-apoptotic genes (*PMAIP1*, *BBC3* and *BCL2L11*) are relative to the pre-treatment levels (0 h) in the bar graph (n = 3). *: p < 0.05; **: p < 0.01; ***: p < 0.005.


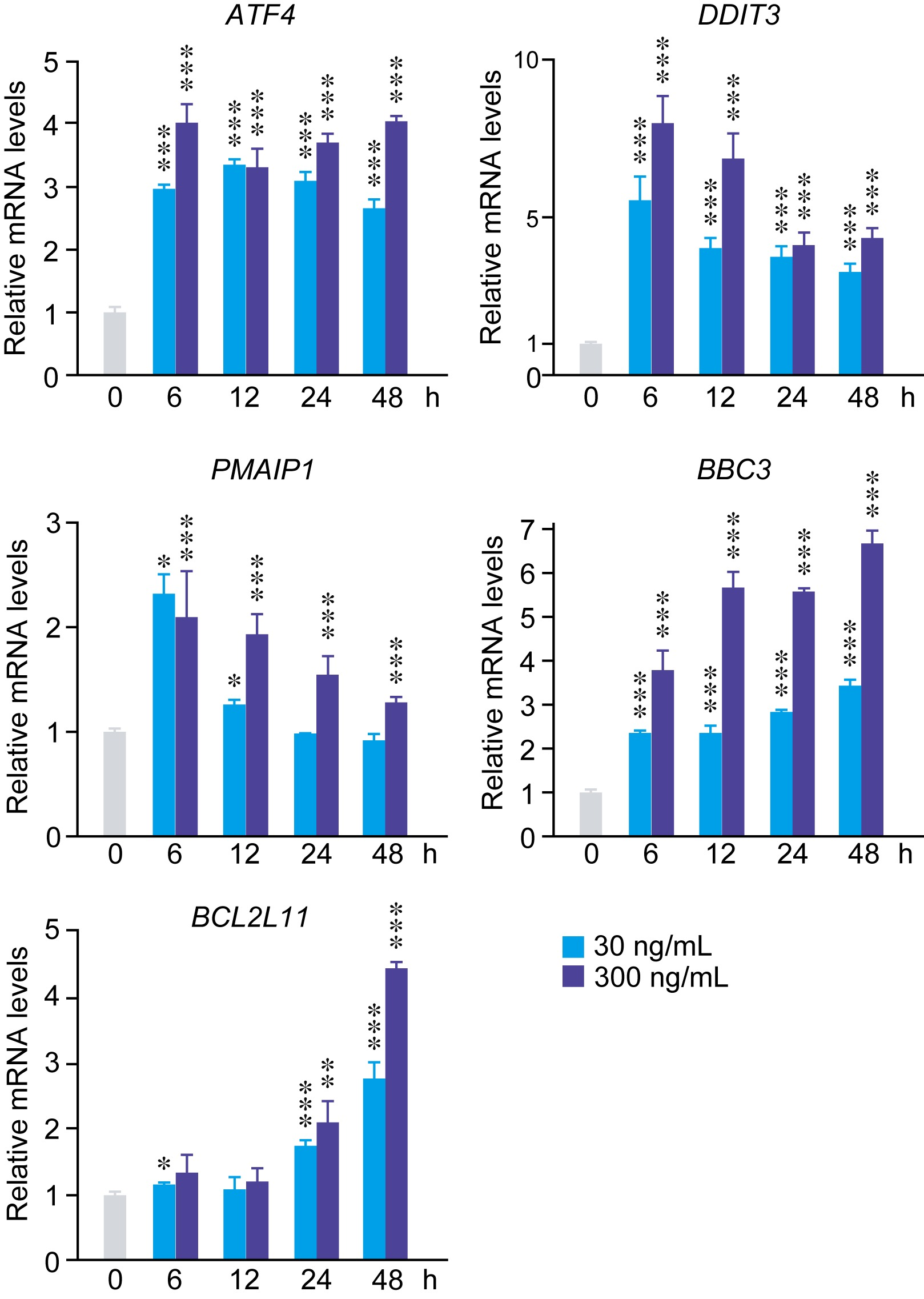

Supplement: S3 Fig — The THP-1 cells were treated with 30 or 300 ng/mL mycolactone for the indicated periods. mRNA levels were analyzed by real-time RT-PCR and normalized to those of GAPDH. The expression levels of ER-stress-related genes (ATF4 and DDIT3) and pro-apoptotic genes (PMAIP1, BBC3 and BCL2L11) are relative to the pre-treatment levels (0 h) in the bar graph (n = 3). *: p < 0.05; **: p < 0.01; ***: p < 0.005. (DOCX) [file pntd.0010672.s003.docx]
