# Supplementary material for: Genome-wide screening identified SEC61A1 as an essential factor for mycolactone-dependent apoptosis in human premonocytic THP-1 cells
Source: PLoS Negl Trop Dis. 2022 Aug 8;16(8):e0010672. doi: 10.1371/journal.pntd.0010672 (PMC9387930; doi:10.1371/journal.pntd.0010672)
Supplement: S4 Fig — Molecular weight markers were included when available. (DOCX) [file pntd.0010672.s004.docx]

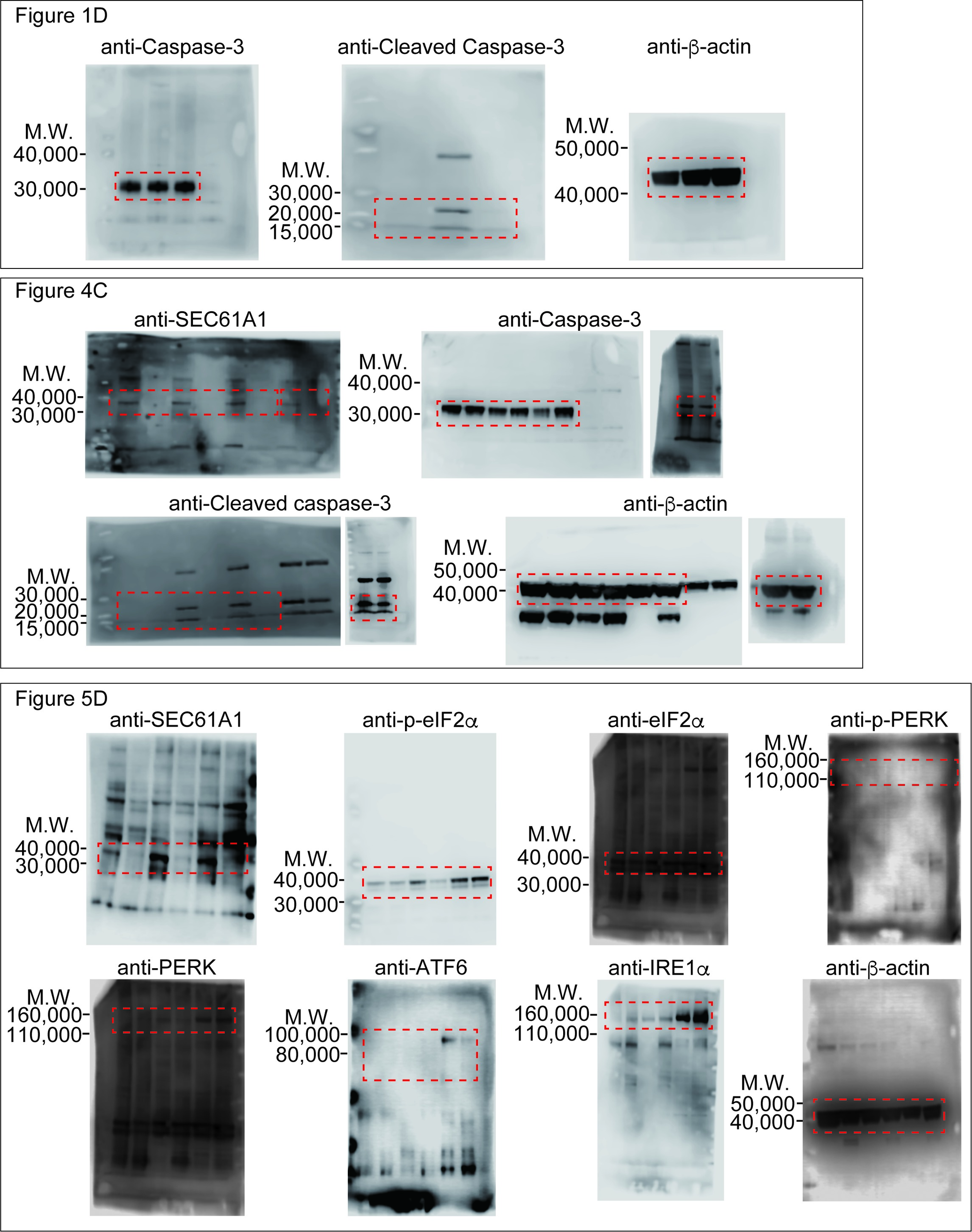
**S4 Fig. Original uncropped images of Western blots from Figs 1, 4, and 5.** Molecular weight markers were included when available.
