# Supplementary material for: Genome-wide screening identified SEC61A1 as an essential factor for mycolactone-dependent apoptosis in human premonocytic THP-1 cells
Source: PLoS Negl Trop Dis. 2022 Aug 8;16(8):e0010672. doi: 10.1371/journal.pntd.0010672 (PMC9387930; doi:10.1371/journal.pntd.0010672)
Supplement: S1 Table — (DOCX) [file pntd.0010672.s005.docx]

**S1 Table.** sgRNA sequences for each target gene.

| Gene | Forward (5'–3') | Reverse (5'–3') |
| --- | --- | --- |
| *EGFP* | CACCGGAGCTGGACGGCGACGTAAA | AAACTTTACGTCGCCGTCCAGCTCC |
| *SEC61A1* | CACCGTCTATCGTGTATGTGATGAC | AAACGTCATCACATACACGATAGAC |
| *ZNF645* | CACCGTGTCACCTTGTCCACCAACG | AAACCGTTGGTGGACAAGGTGACAC |
| *IPO5* | CACCGACCATCATTGCTAACATCTG | AAACCAGATGTTAGCAATGATGGTC |
| *CLEC12A* | CACCGTATGTAGAACCAAGCTGCAC | AAACGTGCAGCTTGGTTCTACATAC |
| *SULT1A4* | CACCGTCCCTCTCCAGGCACCACCT | AAACAGGTGGTGCCTGGAGAGGGAC |
| *R3HDML* | CACCGTGAAATCCAACAAGTTCACG | AAACCGTGAACTTGTTGGATTTCAC |
| *RNF141* | CACCGAGTAGCAAAACATGTTACGT | AAACACGTAACATGTTTTGCTACTC |
| *TNFR11* | CACCGACACACGACAACATATGTTC | AAACGAACATATGTTGTCGTGTGTC |
| *ANKRD33* | CACCGTGAGCAGGGCCACAACACTC | AAACGAGTGTTGTGGCCCTGCTCAC |
| *SGSH* | CACCGTAGTGATGTTCCGCCCCACC | AAACGGTGGGGCGGAACATCACTAC |
