# Supplementary material for: Genome-wide screening identified SEC61A1 as an essential factor for mycolactone-dependent apoptosis in human premonocytic THP-1 cells
Source: PLoS Negl Trop Dis. 2022 Aug 8;16(8):e0010672. doi: 10.1371/journal.pntd.0010672 (PMC9387930; doi:10.1371/journal.pntd.0010672)
Supplement: S2 Table — (DOCX) [file pntd.0010672.s006.docx]

**S2 Table.** Primers used for real-time PCR.

| Gene | Forward (5'–3') | Reverse (5'–3') |
| --- | --- | --- |
| *ATF4* | GGGACAGATTGGATGTTGGAGA | ACCCAACAGGGCATCCAAGT |
| *DDIT3* | AATCAGAGCTGGAACCTGAGGA | TGCTTTCAGGTGTGGTGATGTATG |
| *BCL2L11* | ATGTCTGACTCTGACTCTCG | CTTGTGGCTCTGTCTGTAG |
| *PMAIP1* | GCAGAGCTGGAAGTCGAGTG | GAGCAGAAGAGTTTGGATATCAG |
| *BBC3* | GACGACCTCAACGCACAGTA | AGGAGTCCCATGATGAGATTGT |
| *Spliced XBP1* | GCTGAGTCCGCAGCAGGT | CTGGGTCCAAGTTGTCCAGAAT |
| *Unspliced XBP1* | CAGACTACGTGCACCTCTGC | CTGGGTCCAAGTTGTCCAGAAT |
| *Total*  *XBP1* | TGAAAAACAGAGTAGCAGCTCAGA | CCCAAGCGCTGTCTTAACTC |
| *GAPDH* | TGCACCACCAACTGCTTAGC | GGCATGGACTGTGGTCATGAG |
