# Supplementary material for: Genome-wide screening identified SEC61A1 as an essential factor for mycolactone-dependent apoptosis in human premonocytic THP-1 cells
Source: PLoS Negl Trop Dis. 2022 Aug 8;16(8):e0010672. doi: 10.1371/journal.pntd.0010672 (PMC9387930; doi:10.1371/journal.pntd.0010672)
Supplement: Attachment — Submitted filename: Suzuki K-Mycolactone response220519AK.docx [file pntd.0010672.s008.docx]

**PLOS Neglected Tropical Diseases**

**Response to Reviewers’ Questions**

Reviewer #1: No major new analyses/experiments are required to make the study acceptable for publication. I have, however, suggested two additional experiments that would enhance the novelty of the study and further our understanding of the mechanism of mycolactone-induced cell death in THP-1 cells (see final paragraph of the Summary and General Comments section). Both suggestions are potentially beyond the scope of the current study so, provided that the authors address the points that I have listed in the 'Editorial and Data Presentation Modifications' section, I recommend that the study is acceptable for publication with 'minor revisions', even if the suggested experiments are not performed. This is because the current methods and analyses answer the stated objectives, have been performed with scientific rigor and used correctly to support the current conclusions.

**Response:**

We thank the reviewer for the kind comments and valuable feedback. According to the suggestion, we have performed additional experiments. We have responded to each comment of the reviewers individually as described below.

Reviewer #2: The methods are fully described and appropriate to the objectives of the study. The statistics are sufficient to support the conclusions. There are no ethical or regulatory concerns.
Reviewer #3: The work was carried out with the appropriate methodology.

**Response:**

We thank the reviewers for their careful work and helpful suggestions.

**Results**
Reviewer #1: Presented analysis matches the analysis plan.
Results are clearly and completely presented, and figures are clear. Minor errors: a spelling mistake in Figure 2 and the implication that XBP1 was identified as candidate in the text but not in any of the Figures, Table 1 or source data (more detail given in the Editorial and Data Presentation Modifications section).

**Response:**

We would like to thank the reviewer for pointing out these errors in the manuscript. Regarding XBP1, the following sentence in the **Discussion** section was deleted: **(page 25, lines 428-429 of the original manuscript)**

*“XBP1, encoding a sensor and transducer of ER stress [46]“*

Reviewer #2: The data are very well presented, and all figures and legends are of high quality.

**Response:**

We appreciate your positive comments.

Reviewer #3: I am happy with presentation of the results.

**Response:**

We appreciate your positive comments.
--------------------

**Conclusions**
Reviewer #1: Conclusions are supported by the presented data.
Limitations are described and the authors discuss how these data can advance our understanding of mycolactone-induced cell death but do not place this in the context of current literature where these mechanisms have been elucidated in significant detail using other cell lines (Ogbechi et al. 2018, Morel et al. 2018).
Public health relevance is briefly discussed but lacks clarity.

**Response**:

According to the suggestion, we have mentioned the public health relevance of inhibiting mycolactone activity in the **Discussion** section**.**

“Several approaches have been proposed to inhibit mycolactone activity, *e.g.,* the use of IgG antibody against mycolactone [57, 58], the inhibition of the mycolactone synthesis pathway with substrates (malonyl-CoA, methyl malonyl-CoA) [59] and the attenuation of cytotoxicity with antioxidants [48, 49]. At present there is no effective drug to reduce the toxicity of mycolactone, and further drug screening and analysis of the biological activity of mycolactone will be required to develop novel therapeutic modalities to inhibit mycolactone-induced ulceration.” **(Page 32, lines 573-579)**

Reviewer #2: I have several reservations about the conclusions drawn from this data and feel more work is needed to support the authors' interpretation of the results, mostly related to conclusion that the cell death caused by mycolactone is entirely due to the induction of ER stress.

**Response**:

To address the reviewer’s concern, we further assessed the ER stress response pathway, IRE1α-*XBP1* splicing, PERK-eIF2α phosphorylation and ATF6 cleavage using real-time PCR and immunoblotting. We demonstrated that mycolactone induced *XBP1* splicing and eIF2α phosphorylation, but not ATF-6 cleavage in THP-1 cells. We also demonstrated that eIF2α phosphorylation was suppressed by *SEC61A1* knockout, suggesting that mycolactone-induced eIF2α phosphorylation is mediated by SEC61A1. We have added the new data about ER stress responses in **Fig 5C and D** and discussed the results in the **Results** section.

**“**The ER stress response is triggered through three pathways, *i.e.,* IRE1-*X-box binding protein 1 (XBP1)* splicing, PERK-eIF2α phosphorylation and ATF6 cleavage [38, 39]. When ER stress is activated, IRE1α splices the *XBP1* mRNA, producing an active transcription factor that stimulates the ER stress response [38, 39]. We therefore performed real-time PCR to evaluate the mRNA levels of these genes. *XBP1* splicing was induced in 3 h, peaked at 6 h and decreased in 24 h in *EGFP*-knockout cells (Fig 5C), suggesting that mycolactone activated the IRE1α-*XBP1* pathway. However, *SEC61A1*-knockout cells showed higher levels of *XBP1* splicing even before mycolactone treatment (Fig 5C).

Next, we analyzed the phosphorylation of proteins involved in the ER stress pathway by Western blotting. The eIF2α phosphorylation was induced by mycolactone in *EGFP*-knockout cells, but not in *SEC61A1*-knockout cells (Fig 5D). The PERK protein showed a weak signal, and its phosphorylation was not evident in THP-1 cells. Although full-length ATF6 was not detected in THP-1 cells, thapsigargin, an inducer of ER stress, induced ATF6 in *EGFP*-knockout cells but not in *SEC61A1*-knockout cells. Thapsigargin also induced IRE1α and eIF2α phosphorylation in all these cells. This evidence suggests that *SEC61A1* knockout affects ATF6 protein synthesis. These results also indicate that mycolactone induced *XBP1* splicing and eIF2α phosphorylation in THP-1 cells. Cells with the *SEC61A1* deletion showed sustained *XBP1* splicing but were resistant to mycolactone treatment (Fig 5C and S2 Fig D). Taken together, these results suggest that *SEC61A1* is an essential factor mediating the ability of mycolactone to induce eIF2α phosphorylation.**” (Page 24, lines 432-453)**

We also discussed ER stress response induced by mycolactone in the **Discussion** section.

**“**Mycolactone induced the ER stress response, which triggers cell apoptosis [15, 46]. ER stress responses are activated by three major pathways including IRE1-*XBP1* splicing, phosphorylation of eIF2α via multiple kinases (PERK, general control non-repressed 2: GCN2, protein kinase RNA-activated: PKR), and ATF6 cleavage. Each pathway activates a pro-survival response, responsible for restoring normal ER function by reducing unfolded proteins [56]. Finally, failure to remove stress results in the expression of proapoptotic signaling genes such as *PMAIP1*, *BBC3* and *BCL2L11*.

In this paper, we showed that mycolactone induced IRE1-*XBP1* splicing and eIF2α phosphorylation, but not ATF6 cleavage. It was reported that mycolactone induced *XBP1* splicing in MutuDCs [46] and eIF2α phosphorylation in HeLa cells and MEF cells [15]. We also demonstrated that mycolactone induced ER stress responses in THP-1 cells. The *SEC61A1* deletion cells have diminished eIF2α phosphorylation and expression of proapoptotic genes. Although phosphorylation of PERK, one of the eIF2α kinases, was not detected in THP-1 cells, other kinases such as GCN or PKR may phosphorylate eIF2α with mycolactone treatment [15]. In the present study, the ER stress inducer thapsigargin induced eIF2α phosphorylation in *EGFP*- and *SEC61A1*-knockout THP-1 cells. ATF6 was upregulated by thapsigargin in control, but not in *SEC61A1*-knockout cells. Therefore, it is possible to infer that thapsigargin induces eIF2α phosphorylation independent of SEC61A1, whereas ATF6 expression was dependent on SEC61A1. It was reported that the SEC61A1 inhibitor ipomoeassin F also suppressed ATF6 protein expression in HepG2 cells [9]. Morel *et al*. demonstrated that mycolactone induced an atypical ER stress response, in which the ATF4/DDIT3 branch of the ER stress response was robustly activated, but *Bip*, an upstream regulator of the ER stress response, was downregulated [39, 46]. It was different from the conventional ER stress response induced by thapsigargin, tunicamycin, and MG132. Our study also showed mycolactone-induced *ATF4/DDIT3* expression, which was diminished by *SEC61A1* knockout. *ATF6* gene activation was induced only by thapsigargin and not by mycolactone. We have confirmed that the SEC61A1 knockout suppressed mycolactone-induced ER stress and cytotoxicity.**”** **(page 30, lines 540-568)**

To address the reviewer’s concern that the cell death caused by mycolactone may not be entirely due to the induction of ER stress, we assessed the ER stress response in *EGFP-*knockout and *SEC61A1-*knockout cells. Although we have demonstrated that SEC61A1 mediated mycolactone-induced eIF2α phosphorylation **(Fig 5D)**, the viability assay and FLICA assay have shown that *SEC61A1* knockout partly blocked mycolactone-induced cell death in 144 h **(Fig 3)**. Therefore, other mycolactone-induced stress pathways may also be involved in inducing apoptosis in THP-1 cells. We have discussed this issue in the **Discussion** section.

**"**However, the viability assay and FLICA assay demonstrated that the SEC61A1 knockout was not completely prevented by mycolactone-induced apoptosis. This evidence suggests that other mycolactone-induced stress pathways may also be involved in inducing apoptosis in THP-1 cells*.* Further study is required to demonstrate the precise molecular mechanisms of SEC61A1 in ER stress apoptosis.**” (page 31, lines 568-572)**

Reviewer #3: The conclusions are well supported by the data. However, limitations of the study are not currently sufficiently discussed especially with regard to the known role of Sec61 in mediating mycolactone cytotoxicity. This needs to be corrected in a revised manuscript.

**Response:**

According to the suggestion, we have elaborated on the previously reported cytotoxicity through SEC61A1 induced by mycolactone as simply as possible in the **Discussion** section.

“Thus, our results corroborate previous studies using mutation screening and structural analysis, which identified SEC61A1 as one of the targets of mycolactone action [11, 13, 15, 40].” **(page 28, lines 480-482)**
--------------------

Editorial and Data Presentation Modifications

Reviewer #1: Line 118 vs. 247: Z-DEVD-FMK is described as ‘a specific and irreversible caspase-3 inhibitor’ (line 118) and a ‘pan-caspase inhibitor’ (line 247). Z-DEVD-FMK shows potent inhibition of other caspases so is not a ‘specific’ inhibitor of caspase-3 (please amend in line 118). Since Z-DEVD-FMK also inhibits caspase-8 (which acts upstream of caspase-9 and caspase-3) in cells, perhaps it would be more appropriate to say that ‘mycolactone induced caspase-dependent apoptosis in THP-1 cells’ (line 254) rather than being specific for caspase-3?

**Response:**

We would like to thank the reviewer for pointing out the errors we had overlooked. The Z-DEVD-FMK peptide also inhibits caspase-7, 8 and 9, which are activated by cytochrome c and dATP (Berger *et al*., 2006 PMID: 17117159). We have cited this reference (reference 21) and amended the text to mention Z-DEVD-FMK as a pan-caspase inhibitor in the **Methods** section.

*“*Thapsigargin (Wako) was used to induce ER stress as a control. z-Asp-Glu-Val-Asp-fluoromethyl ketone (Z-DEVD-FMK), a pan-caspase inhibitor, was purchased from Cayman Chemical (Ann Arbor, MI, USA). The DEVD-FMK peptide inhibits caspase-3,7, 8 and 9 [21, 22].” **(page 7, lines 133-136)**

Line 301: spelling error in Fig. 2A, step 4; change ‘mycoractone’ to ‘mycolactone’. 

**Response:**

The misspelling was corrected. Thank you for the comment **(Fig. 2A).**

Line 330: Was there a ‘no treatment’ control for any of the sgRNA treated cells? Or have the values shown here been expressed relative to the ‘no treatment’ for each sgRNA? For example, I’d expect the viability of SEC61A1-KO cells to start declining after 96-144 h even when not treated with mycolactone?

**Response:**

We have omitted each value of sgRNA treated THP-1 cells without mycolactone in **Fig 3**. The cell viability of each sgRNA-treated THP-1 cell without mycolactone is 100% by the trypan blue exclusion assay. *SEC61A1* knockout cells did not decline in our study. *SEC61A1-*knockout THP-1 cells were selected with 0.5 μg/mL puromycin for 1 week. The surviving cells were reseeded and expanded to a large enough number to experiment for 2 weeks. *SEC61A1*-knockout cells have low growth rates but can survive without a declining cell number. We have added details of the puromycin selection of sgRNA-treated cells in the **Methods** section**.**

**"**THP-1 cells were incubated with lentiviral vectors for 16 h in the presence of 8 μg/mL polybrene (Sigma-Aldrich) for lentivirus transduction, then selected with 0.5 μg/mL puromycin (Sigma-Aldrich) for at least 1 week. Surviving cells were reseeded in a 10 cm dish and expanded to use for the experiments.” **(page 10, lines 193-197)**

We also discussed the viability and growth of *SEC61A1*-knockout cells in the **Discussion** section**.**

“SEC61A1 has an important role in protein translocation and calcium leakage [42, 46]. It was reported that *SEC61A1* deletion or silencing resulted in growth defects or cell death in association with sustained *XBP1* splicing [34, 35, 50]. In our study, deletion of *SEC61A1* showed a low growth phenotype and sustained *XBP1* splicing in THP-1 cells (Fig 5C and S2 Fig D). However, *SEC61A1* deletion did not induce apoptosis or the expression of proapoptotic genes such as *PMAIP1*, *BBC3* and *BCL2L11* (Fig 5B). We could not elucidate the mechanism contributing to the different growth and cytotoxic phenotype of *SEC61A1* deletion between THP-1 cells and other cells. In our genome-wide screening, *SEC61A1*-knockout cells had low growth rates but could survive in the presence of mycolactone; *SEC61A1* was found as a top score gene (Table 1).” **(page 29, lines 514-524)**

Line 336: Reference(s) for FLICA probe?

**Response:**

We have added the reference (Bender et al. 2000; PMID: 29915147, reference 22) for the FLICA probe in the **Methods** section**.**

“Activation of caspase-dependent apoptosis activity was analyzed using the fluorochrome-labeled inhibitors of caspases (FLICA) Caspase-3/7 Assay Kit (Immunochemistry Technologies, Bloomington, MN, USA) according to the manufacturer’s instructions [22].” **(page 13, lines 259-262)**

Line 340: Could benefit from stating that Actinomycin D (ActD) is a transcription inhibitor ie. to make it clearer that there is no difference in apoptosis induction between the EGFP- and SEC61A1-KO cells.

**Response:**

According to the suggestion, we have mentioned that cell death induced by actinomycin D is independent of the ER stress pathway in the **Result** section.

“ActD is a transcription inhibitor which intercalates into DNA to inhibit new mRNA synthesis, arrest the cell cycle and induce caspase-3 dependent apoptosis [36], independent of ER stress. As a result, the knockout of *SEC61A1* had no effect on ActD-induced caspase-3 activation following mycolactone treatment (Fig 4C).” **(page 21, lines 393-397)**.

Line 401: Suggest text is amended to: ‘which are known to trigger cytochrome c release from…’ It currently sounds like this has been experimentally determined in this manuscript when it hasn’t.

**Response:**

We have amended the sentence as suggested in the **Discussion** section.

“Since SEC61A1 is a component of the ER translocon, we additionally showed that mycolactone activates the expression of ER stress-related genes, which are known to trigger cytochrome c release from mitochondria to activate the caspase cascade and thus apoptosis [41].” **(page 28, lines 482-487)**.

Line 426-431: The authors include XBP1 as one of the genes identified as a candidate of mycolactone-induced cell death with a lower RRA value than SEC61A1 and state that knockout of these genes did not alter mycolactone-induced apoptosis in THP-1 cells. However, there is no mention of XBP1 in any of the Figures, Table 1 or source data.

**Response:**

We would like to thank the reviewer for pointing out our errors in the manuscript. Regarding XBP1, the following sentence in the Discussion section was deleted: **(page 25, lines 428-429 of the original manuscript)**

*“XBP1, encoding a sensor and transducer of ER stress [46]“*

Line 441: Note spelling error; change ‘dependant’ to ‘dependent’. 

**Response:**

We have corrected ‘dependant’ to ‘dependent’. Thank you for the comment (line 482 of the revised manuscript).

Line 441-447: This section lacks clarity. What is meant by ‘inhibition of mycolactone’? Do you mean inhibit its biogenesis by M. ulcerans or something else? Could you expand on how Sec61alpha could be inhibited with reduced toxicity than that induced by mycolactone-mediated inhibition of Sec61? Do you think that a more substrate-selective Sec61 inhibitor (eg. the cotransin derivative CT8; see Pauwels et al. 2021) could be used to compete with mycolactone-Sec61 binding during an M. ulcerans infection?

**Response:**

Thank you for pointing out this important issue. We meant “inhibition of mycolactone activity”, but it was not clear in the text. To clarify the reviewer’s concern, we have modified the **Discussion** section**.**

“Several approaches have been proposed to inhibit mycolactone activity, *e.g.,* the use of IgG antibody against mycolactone [57, 58], the inhibition of the mycolactone synthesis pathway with substrates (malonyl-CoA, methyl malonyl-CoA) [59] and the attenuation of cytotoxicity with antioxidants [48, 49]. At present there is no effective drug to reduce the toxicity of mycolactone, and further drug screening and analysis of the biological activity of mycolactone will be required to develop novel therapeutic modalities to inhibit mycolactone-induced ulceration.” **(page 32, lines 573-579)**

“Since mycolactone is the sole pathologic factor of *M. ulcerans*, inhibition of mycolactone activity or its downstream cascades could be a novel therapeutic modality to eliminate the harmful effects of mycolactone, in addition to the 8-week antibiotic regimen of rifampicin and clarithromycin.” **(page 32, lines 582-585).**

Line 541: The authors acknowledge ‘synthetic and biotinylated mycolactone A/B’ but biotinylated mycolactone is not mentioned elsewhere in the manuscript?

**Response:**

We thank the reviewer for this comment. In this paper we use only synthetic mycolactone A/B, not biotinylated mycolactone. We have deleted “biotinylated” and modified “synthetic mycolactone A/B” in the **Acknowledgement** section**.**

“We are grateful to Dr. Yoshito Kishi of Harvard University for the kind donation of synthetic mycolactone A/B.” **(page 33, lines 591-592)**

Reviewer #2: There are some errors in interpretation of current knowledge that need to be corrected.

1)	In line 407, the authors state that calcium leakage from the ER is “required for stabilization of protein folding” and in line 421 suggest that mycolactone inhibits calcium leakage. Calcium leakage is an inevitable consequence of the opening of the Sec61 channel. Countering this to maintain ER calcium levels is the process required for protein folding and cells go to some lengths to minimise the leak via BiP in the ER lumen and calmodulin in the cytosol.  Recent evidence suggests that mycolactone does not inhibit the leak but actually increases it (see Bhadra et al, 2021, PMID: 34726690). This needs to be discussed.

**Response:**

Thank you for pointing out our mistake. We have mentioned this issue in the revised manuscript and cited the work of Bhadra et al. (reference 44).

“Mycolactone alters the Sec61 translocon conformation to open the cytosolic side of the lateral gate and enhance calcium leakage [44].” **(pages 29, lines 500-502)**

2)	In line 420 the authors speculate that “mycolactone directly binds to SEC61A1, thereby inhibiting modification of signalling peptides … which results in accumulation of unfolded proteins in the ER” and in line 446 they suggest that the effects of mycolactone may be blocked by inhibiting SEC61A1. It is now well established that mycolactone is in fact an inhibitor of SEC61A1 (see refs 29 and 30 for example). As mycolactone prevents proteins passing through the translocon, the authors need to address how it might cause accumulation of unfolded proteins within the ER.

Finally, most of the other known inhibitors of SEC61A1 activity induce a remarkably similar phenotype to mycolactone (eg Ipomoeassin F, see Zong et al, 2019, PMID: 31059257 and Roboti et al, 2021, PMID: 34079010) and therefore would be expected to mimic the effects of mycolactone rather than protect against them. These parts of the discussion need to be rewritten.

**Response:**

Thank you for pointing out this important issue. We modified the text to mention that mycolactone binds SEC61A1 and induces calcium leakage and mislocated protein accumulation in the **Discussion** section.

“Therefore, mycolactone directly binds to SEC61A1 thereby inhibiting the modification of signaling peptides and enhancing calcium leakage, which results in the accumulation of mislocated proteins [8, 46, 47].” **(pages 29, lines 507-510)**.

We also discussed the action of other inhibitors and the work of Zong et al. (reference 9) and Roboti et al (reference 12).

“*In vitro* studies showed that mycolactone blocks the translocation of nascent proteins across the ER membrane, and similar inhibitors such as CT7, CT8, and ipomoeassin F directly target the ER Sec61 translocon [7-12].” **(pages 5-6, lines 93-95)**.

Reviewer #3: I would recommend considering this manuscript if my concerns can be adequately addressec.

**Response:**

Thank you for the kind comment. We have addressed all the concerns raised by the reviewer and have modified the manuscript. We believe that the modification has strengthened the manuscript.
--------------------

Summary and General Comments

Reviewer #1: The manuscript by Kawashima et al. focusses on understanding the genes necessary for mycolactone-induced cell death in human premonocytic THP-1 cells. Mycolactone is an exotoxin produced by Mycobacterium ulcerans, the causative agent of the necrotising skin disease known as Buruli ulcer, and it is well established that the pathology of this disease is directly linked to mycolactone binding to the central subunit of the Sec61 complex, Sec61alpha (Demangel and High 2018). The Sec61 complex is the predominant protein conducting channel via which secretory and transmembrane proteins traverse, or are inserted into, the membrane of the endoplasmic reticulum (ER) (O’Keefe et al. 2021). By binding to the cytosolic side of Sec61alpha and stabilising a partially open conformation of Sec61 (Gérard et al. 2020), mycolactone precludes the entry of most newly synthesised polypeptides into the Sec61 channel and, hence, inhibits their access to the secretory pathway. This global blockade in ER protein translocation reduces the ability of cells to synthesise many secretory and transmembrane proteins (type I, type II but not type III or tail-anchored proteins; McKenna et al. 2017, Morel et al. 2018, Zong et al. 2019), triggering stress responses that ultimately induce apoptosis and cell death (Ogbechi et al. 2018, Morel et al. 2018).

Here, the authors first use trypan blue and Hoescht 33342 staining, together with the pan-caspase inhibitor Z-DEVD-FMK, to show that mycolactone-induced cell death proceeds via a caspase-3 dependent apoptotic pathway in THP-1 cells. They then use genome-wide CRISPR/Cas9-mediated screening to identify 884 candidate genes involved in mycolactone-induced cell death. After sgRNA-mediated knockout of each of the top 10 scored candidate genes, the authors find that only SEC61A1-KO THP-1 cells showed prolonged survival when treated with mycolactone. Next, by using a fluorescent probe that binds to activated caspase-3, the small molecule apoptosis inducer Actinomycin D and western blot analysis, the authors show that Sec61alpha is required for mycolactone-induced caspase-3 activation. Finally, the authors use real-time PCR to show that mycolactone-treated THP-1 cells induce mRNA expression of the ER-stress related genes ATF4 and DDIT3 (CHOP) after 6 h and, after prolonged exposure,
increased mRNA levels of the pro-apoptotic genes PMAIP1 (Noxa), BBC3 (Puma) and BCL2L11 (Bim); effects that are ablated in SEC61A1-KO cells. Taken together, the authors conclude that Sec61alpha is essential for the ability of mycolactone to induce ER stress and caspase-3 dependent apoptosis. From this, they propose that either inhibiting Sec61alpha or downstream ER stress and caspase-3 dependent apoptotic factors presents a novel approach to eliminate the harmful effects of mycolactone during M. ulcerans infection.

Overall, this is a well-executed and technically sound study. However, despite the use of sophisticated biochemical methods and genome-wide screening, most of what is shown here confirms previous work which has already established that mycolactone’s toxicity strictly depends on its binding to Sec61alpha (Baron et al. 2016) and that ATF4 and CHOP mRNA expression is increased in response to mycolactone in RAW264.7 (Ogbechi et al. 2018), HeLa (Ogbechi et al. 2018) and dendritic (Morel et al. 2018) cells. 

The present study modestly extends this work by demonstrating that, in THP-1 cells, mycolactone-treatment increases the mRNA levels of ATF4, CHOP, PMAIP1 (Noxa), BBC3 (Puma) and BCL2L11 (Bim) and induces caspase-3 dependent apoptosis. Whether this caspase-dependent apoptosis is the result of; i) a mycolactone-induced integrated stress response in the absence of the UPR (RAW264.7 and HeLa cells; Ogbechi et al. 2018); ii) an atypical UPR as observed in mycolactone-treated dendritic cells (Morel et al. 2018) and ipomoeassin-F-treated HepG2 cells (Roboti et al. 2021) or iii) a combination of stress responses in THP-1 cells, would certainly enhance our understanding of mycolactone-induced cell death in different cell types. The authors could potentially address this question by monitoring XBP1 mRNA splicing in mycolactone-treated THP-1 cells using real-time or reverse-transcription PCR (cf. Ogbechi et al. 2018, Morel et al. 2018, Roboti et al. 2021) or assessing the level of mRNA induction of ATF4 and/or CHOP in mycolactone-treated ATF4-KO THP-1 cells (cf. Ogbechi et al. 2018). Although these experiments are potentially beyond the scope of the current study, the authors can significantly improve their manuscript by placing their results within the context of other recent studies that have advanced our understanding of the mechanisms of mycolactone-induced apoptosis (eg. Morel et al. 2018, Ogbechi et al. 2018).

In summary, Kawashima et al. set out to identify the genes necessary for mycolactone-induced cell death in human premonocytic THP-1 cells. They successfully achieved their objective and, out of 884 candidate genes identified by genome-wide screening, they identified Sec61A1 as the only essential gene that is required for the mycolactone-mediated induction of caspase-dependent apoptosis in THP-1 cells.

**Response:**

We thank the reviewer for the careful review and detailed suggestions. According to the suggestions, we performed additional experiments about the ER stress response. We demonstrated that mycolactone induced ER stress responses such as XBP-1 splicing and eIF2α phosphorylation. We showed that *SEC61A1* knockout resulted in suppression of eIF2α phosphorylation and apoptosis. Additional data were shown in **Fig 5C and D.**

“The ER stress response is triggered through three pathways, *i.e.,* IRE1-*X-box binding protein 1 (XBP1)* splicing, PERK-eIF2α phosphorylation and ATF6 cleavage [38, 39]. When ER stress is activated, IRE1α splices the *XBP1* mRNA, producing an active transcription factor that stimulates the ER stress response [38, 39]. We therefore performed real-time PCR to evaluate the mRNA levels of these genes. *XBP1* splicing was induced in 3 h, peaked at 6 h and decreased in 24 h in *EGFP*-knockout cells (Fig 5C), suggesting that mycolactone activated the IRE1α-*XBP1* pathway. However, *SEC61A1*-knockout cells showed higher levels of *XBP1* splicing even before mycolactone treatment (Fig 5C).

Next, we analyzed the phosphorylation of proteins involved in the ER stress pathway by Western blotting. The eIF2α phosphorylation was induced by mycolactone in *EGFP*-knockout cells, but not in *SEC61A1*-knockout cells (Fig 5D). The PERK protein showed a weak signal, and its phosphorylation was not evident in THP-1 cells. Although full-length ATF6 was not detected in THP-1 cells, thapsigargin, an inducer of ER stress, induced ATF6 in *EGFP*-knockout cells but not in *SEC61A1*-knockout cells. Thapsigargin also induced IRE1α and eIF2α phosphorylation in all these cells. This evidence suggests that *SEC61A1* knockout affects ATF6 protein synthesis. These results also indicate that mycolactone induced *XBP1* splicing and eIF2α phosphorylation in THP-1 cells. Cells with the *SEC61A1* deletion showed sustained *XBP1* splicing but were resistant to mycolactone treatment (Fig 5C and S2 Fig D). Taken together, these results suggest that *SEC61A1* is an essential factor mediating the ability of mycolactone to induce eIF2α phosphorylation.” **(page 24, lines 432-453)**

We also modified the manuscript to place our results in the context of other recent studies that have advanced our understanding of the mechanisms of mycolactone-induced apoptosis with the references suggested by the reviewer. The following new references have been added: Morel et al. 2018; PMID: 29915147 (reference 46), and Roboti et al. 2021; PMID: 34079010 (reference 9)**.**

“In this paper, we showed that mycolactone induced IRE1-*XBP1* splicing and eIF2α phosphorylation, but not ATF6 cleavage. It was reported that mycolactone induced *XBP1* splicing in MutuDCs [46] and eIF2α phosphorylation in HeLa cells and MEF cells [15]. We also demonstrated that mycolactone induced ER stress responses in THP-1 cells. The *SEC61A1* deletion cells have diminished eIF2α phosphorylation and expression of proapoptotic genes. Although phosphorylation of PERK, one of the eIF2α kinases, was not detected in THP-1 cells, other kinases such as GCN or PKR may phosphorylate eIF2α with mycolactone treatment [15]. In the present study, the ER stress inducer thapsigargin induced eIF2α phosphorylation in *EGFP*- and *SEC61A1*-knockout THP-1 cells. ATF6 was upregulated by thapsigargin in control, but not in *SEC61A1*-knockout cells. Therefore, it is possible to infer that thapsigargin induces eIF2α phosphorylation independent of SEC61A1, whereas ATF6 expression was dependent on SEC61A1. It was reported that the SEC61A1 inhibitor ipomoeassin F also suppressed ATF6 protein expression in HepG2 cells [9]. Morel *et al*. demonstrated that mycolactone induced an atypical ER stress response, in which the ATF4/DDIT3 branch of the ER stress response was robustly activated, but *Bip*, an upstream regulator of the ER stress response, was downregulated [39, 46]. It was different from the conventional ER stress response induced by thapsigargin, tunicamycin, and MG132. Our study also showed mycolactone-induced *ATF4/DDIT3* expression, which was diminished by *SEC61A1* knockout. *ATF6* gene activation was induced only by thapsigargin and not by mycolactone. We have confirmed that the SEC61A1 knockout suppressed mycolactone-induced ER stress and cytotoxicity.” **(page 31, lines 547-568)**

Reviewer #2: This work describes an investigation into the mechanism of cell death induced by the Buruli Ulcer virulence factor mycolactone using genome scale CRISPR screen in THP-1 cells. The authors found and validated SEC61A1 as a candidate gene. Knockout of SEC61A1 protected cells against mycolactone-induced cytotoxicity, prevented caspase 3 activation and reduced expression of stress related and pro-apoptotic genes. This well written and interesting paper contributes to our understanding of mycolactone’s activity. However, as well as needing further experiments to back up the conclusions, there are some omissions in the document the weaken the overall impact. There is a brief discussion of the results in the context of previously published works but there are many highly relevant publications that have been largely ignored. For example, the widely reported role of oxidative stress in mycolactone cytotoxicity is not mentioned and multiple papers showing inhibition of Sec61 activity by mycolactone are uncited. When comparing their results to other screens, the authors merely suggest that the differences in their findings to previous screens may be due to cell type, but this seems unlikely given the universal expression of SEC61A1 and the essential nature of its activity. The discussion should be expanded to include a more thorough analysis of the literature and the implications of the findings.

Experimentally there are several areas that need more work to justify the authors conclusions.

1)	There are a number of CRISPR-based studies that have identified SEC61A1 as an essential gene and report a severe growth defect in knockout cells (eg Adamson et al, 2016, PMID: 27984733).  Some data on the growth of the knockout THP-1 cells would be useful here as changes in growth may affect cellular responses. Confirmation of SEC61A1 knockout by PCR would also strengthen the authors findings.

**Response:**

We appreciate this comment to help us to improve our manuscript. We have selected stable *SEC61A1*-knockout THP-1 cells with puromycin. In the selection process we removed dead cells and harvested surviving cells. This cell line showed a growth defect**.** We have included the data **(S2 Fig D)** and mentioned this issue in the **Results** section**.**

“After puromycin selection of knockout cells, we compared cell growth in control, *EGFP*- and *SEC61A1*-knockout cells (S2 Fig D). *SEC61A1*-knockout cells grew more slowly than intact THP-1 cells and *EGFP*-knockout cells.” **(page 21, lines 381-383)**

In addition, we performed PCR analysis and DNA sequencing to further confirm *SEC61A1* knockout as suggested by the reviewer. We have added the methods and data with figures **(S2 Fig A, B and C)** in the **Method** section

"**Genome mutation analysis**

To confirm the SEC61A1 genome mutation induced by the SEC61A1-targeting sgRNA, we extracted genomic DNA from *EGFP*-knockout and *SEC61A1*-knockout THP-1 cells using the QIAamp DNA Mini Kit (Qiagen) and subjected the genomic DNA to PCR to amplify the *EC61A1* sgRNA targeting site using the following primers: (sense) 5′- GCCTGGCGTTGAATTGGTG-3′ and (antisense) 5′- AAGTGTGAGGGGCTACTCAA-3′. PCR was performed using the Thermal Cycler Dice (Takara Bio, Shiga, Japan). Briefly, the PCR mixture was first denatured for 5 min at 94 °C, followed by 15 cycles of three-temperature PCR consisting of denaturation for 30 sec at 94 °C, annealing for 30 sec at 65 °C and extension at 72 °C for 45 sec. The PCR products were analyzed by 2% agarose gel electrophoresis. The SEC61A1 targeting site was also analyzed by direct sequencing with (sense) 5′- GCCTGGCGTTGAATTGGTG using the Fasmac sequencing service (Fasmac, Atsugi, Japan).” **(page 12, lines 243-256.)**

In the **Result** section.

“We next explored the possibility that SEC61A1 is involved in mycolactone-induced apoptosis, especially focusing on the molecular mechanisms of apoptosis in THP-1 cells. First, we confirmed SEC61A1 depletion by Western blotting, PCR analysis and DNA sequencing of the sgRNA binding site in *SEC61A1* (Fig S2A, B and C, respectively). Thereafter, we confirmed *SEC61A1* deletion by the CRISPR/Cas9 genome editing system. SEC61A1 is known to be important for protein translocation and calcium leakage, and SEC61A1 blockade affects cell growth and survival [34, 35].” **(page 20, lines 374-380).**

2)	The stress response genes analysed by the authors are not specific for ER-stress and could be induced by other pathways. It cannot be concluded that mycolactone induces ER-stress based on this alone. This is important because Ogbechi et al (Ref 24) showed that mycolactone can activate an integrated stress response via multiple pathways. In addition, Adamson et al (see above) reported that SEC61A1 knockout actually activates the IRE1/XPB1 arm of the ER-stress response. To make a strong case for ER-stress as the major cause of cell death in mycolactone treated cells, the authors need to show all three arms of the stress response are activated by demonstrating EIF2α phosphorylation, XBP1 splicing and ATF6 cleavage. Use of a control inducer of ER-stress that does not depend directly on Sec61 such Tunicamycin or Thapsigargin would help confirm that the protective effect of SEC61A1 knockout is specific for mycolactone.

**Response:**

We thank the reviewer for this comment. According to the suggestion, we demonstrated quantitative real-time PCR for *XBP1* splicing and immunoblotting for ER stress response proteins **(Fig 5C and D)**. We also demonstrated that mycolactone induced *XBP1* splicing and eIF2α phosphorylation, but not ATF6 cleavage. In *SEC61A1*-knockout cells mycolactone-induced eIF2a phosphorylation was diminished and a high level of *XBP1* splicing was sustained **(Fig 5D)**. We used thapsigargin as an ER stress inducer and showed that thapsigargin induced eIF2α phosphorylation in THP-1 cells regardless of *SEC61A1* knockout. We have included the data and mentioned this issue in the **Result** section**.**

**"**The ER stress response is triggered through three pathways, *i.e.,* IRE1-*X-box binding protein 1 (XBP1)* splicing, PERK-eIF2α phosphorylation and ATF6 cleavage [38, 39]. When ER stress is activated, IRE1α splices the *XBP1* mRNA, producing an active transcription factor that stimulates the ER stress response [38, 39]. We therefore performed real-time PCR to evaluate the mRNA levels of these genes. *XBP1* splicing was induced in 3 h, peaked at 6 h and decreased in 24 h in *EGFP*-knockout cells (Fig 5C), suggesting that mycolactone activated the IRE1α-*XBP1* pathway. However, *SEC61A1*-knockout cells showed higher levels of *XBP1* splicing even before mycolactone treatment (Fig 5C).

Next, we analyzed the phosphorylation of proteins involved in the ER stress pathway by Western blotting. The eIF2α phosphorylation was induced by mycolactone in *EGFP*-knockout cells, but not in *SEC61A1*-knockout cells (Fig 5D). The PERK protein showed a weak signal, and its phosphorylation was not evident in THP-1 cells. Although full-length ATF6 was not detected in THP-1 cells, thapsigargin, an inducer of ER stress, induced ATF6 in *EGFP*-knockout cells but not in *SEC61A1*-knockout cells. Thapsigargin also induced IRE1α and eIF2α phosphorylation in all these cells. This evidence suggests that *SEC61A1* knockout affects ATF6 protein synthesis. These results also indicate that mycolactone induced *XBP1* splicing and eIF2α phosphorylation in THP-1 cells. Cells with the *SEC61A1* deletion showed sustained *XBP1* splicing but were resistant to mycolactone treatment (Fig 5C and S2 Fig D). Taken together, these results suggest that *SEC61A1* is an essential factor mediating the ability of mycolactone to induce eIF2α phosphorylation.” **(page 24, lines 432-453)**

In the **Discussion** section.

**“**Mycolactone induced the ER stress response, which triggers cell apoptosis [15, 46]. ER stress responses are activated by three major pathways including IRE1-*XBP1* splicing, phosphorylation of eIF2α via multiple kinases (PERK, general control non-repressed 2: GCN2, protein kinase RNA-activated: PKR), and ATF6 cleavage. Each pathway activates a pro-survival response, responsible for restoring normal ER function by reducing unfolded proteins [56]. Finally, failure to remove stress results in the expression of proapoptotic signaling genes such as *PMAIP1*, *BBC3* and *BCL2L11*.

In this paper, we showed that mycolactone induced IRE1-*XBP1* splicing and eIF2α phosphorylation, but not ATF6 cleavage. It was reported that mycolactone induced *XBP1* splicing in MutuDCs [46] and eIF2α phosphorylation in HeLa cells and MEF cells [15]. We also demonstrated that mycolactone induced ER stress responses in THP-1 cells. The *SEC61A1* deletion cells have diminished eIF2α phosphorylation and expression of proapoptotic genes. Although phosphorylation of PERK, one of the eIF2α kinases, was not detected in THP-1 cells, other kinases such as GCN or PKR may phosphorylate eIF2α with mycolactone treatment [15]. In the present study, the ER stress inducer thapsigargin induced eIF2α phosphorylation in *EGFP*- and *SEC61A1*-knockout THP-1 cells. ATF6 was upregulated by thapsigargin in control, but not in *SEC61A1*-knockout cells. Therefore, it is possible to infer that thapsigargin induces eIF2α phosphorylation independent of SEC61A1, whereas ATF6 expression was dependent on SEC61A1. It was reported that the SEC61A1 inhibitor ipomoeassin F also suppressed ATF6 protein expression in HepG2 cells [9]. Morel *et al*. demonstrated that mycolactone induced an atypical ER stress response, in which the ATF4/DDIT3 branch of the ER stress response was robustly activated, but *Bip*, an upstream regulator of the ER stress response, was downregulated [39, 46]. It was different from the conventional ER stress response induced by thapsigargin, tunicamycin, and MG132. Our study also showed mycolactone-induced *ATF4/DDIT3* expression, which was diminished by *SEC61A1* knockout. *ATF6* gene activation was induced only by thapsigargin and not by mycolactone. We have confirmed that the SEC61A1 knockout suppressed mycolactone-induced ER stress and cytotoxicity. However, the viability assay and FLICA assay demonstrated that the SEC61A1 knockout was not completely prevented by mycolactone-induced apoptosis. This evidence suggests that other mycolactone-induced stress pathways may also be involved in inducing apoptosis in THP-1 cells*.* Further study is required to demonstrate the precise molecular mechanisms of SEC61A1 in ER stress apoptosis.” **(page 30, lines 540-572)**

We also discussed oxidative stress in mycolactone cytotoxicity in the **Discussion** section.

“However, so far the relationship between ER stress and oxidative stress was not confirmed. Mycolactone also induces reactive oxygen species, which affect cytotoxicity [48, 49].“ **(page 29, lines 511-513).**

3)	Related to the above point, the activators of the unfolded protein response, PERK, IRE1 and ATF6 are all Sec61 dependent proteins. These proteins could be downregulated in the knockout THP-1 cells prior to addition of mycolactone, which would explain the reduced response. The levels of each should be confirmed by immunoblotting.

**Response:**

Thank you for pointing out this important issue. We performed Western blotting to demonstrate PERK, IRE1α and ATF6 expression in *SEC61A1*-knockout cells **(Fig 5D).**

**“**Next, we analyzed the phosphorylation of proteins involved in the ER stress pathway by Western blotting. The eIF2α phosphorylation was induced by mycolactone in *EGFP*-knockout cells, but not in *SEC61A1*-knockout cells (Fig 5D). The PERK protein showed a weak signal, and its phosphorylation was not evident in THP-1 cells. Although full-length ATF6 was not detected in THP-1 cells, thapsigargin, an inducer of ER stress, induced ATF6 in *EGFP*-knockout cells but not in *SEC61A1*-knockout cells. Thapsigargin also induced IRE1α and eIF2α phosphorylation in all these cells. This evidence suggests that *SEC61A1* knockout affects ATF6 protein synthesis. These results also indicate that mycolactone induced *XBP1* splicing and eIF2α phosphorylation in THP-1 cells.” **(page 25, lines 441-450)**

In the **Discussion** section**.**

**“**We also demonstrated that mycolactone induced ER stress responses in THP-1 cells. The *SEC61A1* deletion cells have diminished eIF2α phosphorylation and expression of proapoptotic genes. Although phosphorylation of PERK, one of the eIF2α kinases, was not detected in THP-1 cells, other kinases such as GCN or PKR may phosphorylate eIF2α with mycolactone treatment [15]. In the present study, the ER stress inducer thapsigargin induced eIF2α phosphorylation in *EGFP*- and *SEC61A1*-knockout THP-1 cells. ATF6 was upregulated by thapsigargin in control, but not in *SEC61A1*-knockout cells. Therefore, it is possible to infer that thapsigargin induces eIF2α phosphorylation independent of SEC61A1, whereas ATF6 expression was dependent on SEC61A1. It was reported that the SEC61A1 inhibitor ipomoeassin F also suppressed ATF6 protein expression in HepG2 cells [9]. Morel *et al*. demonstrated that mycolactone induced an atypical ER stress response, in which the ATF4/DDIT3 branch of the ER stress response was robustly activated, but *Bip*, an upstream regulator of the ER stress response, was downregulated [39, 46]. It was different from the conventional ER stress response induced by thapsigargin, tunicamycin, and MG132. Our study also showed mycolactone-induced *ATF4/DDIT3* expression, which was diminished by *SEC61A1* knockout. *ATF6* gene activation was induced only by thapsigargin and not by mycolactone.” (**page 31, lines 550-566)**

Reviewer #3: Review of manuscript of Kawashima et al. entitled” Genome-wide screening identified SEC61A1 as an essential factor for mycolactone-dependent apoptosis in human premonocytic THP-1 cells”

This manuscript describes a CRISPR screen to identify factors involved in mycolactone-induced apoptosis in premonocytic THP-1 human cells. The screen identified SEC61A1 as the main hit gene, whose downmodulation was further validated to confer protection from mycolactone-induced cytotoxicity. It is already well established that mycolactone directly binds to the trimeric Sec61 translocon and blocks its protein translocation activity. Since Sec61 is essential for viability of all eukaryotic cells, its inhibition by mycolactone leads to broad cytotoxicity across many mammalian cell lines. The screen described in this manuscript is well carried out and the SEC61A1 hit is validated and new insights into the apoptosis mechanism resulting from Sec61 inhibition are presented. The already well-established role of Sec61 as the direct cellular target of mycolactone somewhat detracts from the general impact of this work. Still, this work is worth publishing given that the below comments are addressed by the authors.

Major points:
1)	It is already quite abundantly demonstrated by previous studies that the direct cellular target of mycolactone A/B responsible for its cytotoxic effects is the Alpha subunit of the Sec61 complex. I believe it is not correct to completely omit this information from the abstract and this should be corrected in the revised manuscript.

**Response:**

We thank the reviewer for this comment. According to the suggestion, we have included this information in the manuscript in the **Abstract** section**.**

**“**Although previous studies have reported that mycolactone targets SEC61A1 based on mutation screening and structural analysis in several cell lines, we have reconfirmed that SEC61A1 is a mycolactone target by genome-wide screening in THP-1 cells.” **(page 3, lines 45-48)**

In the **Discussion** section**.**

“Thus, our results corroborate previous studies using mutation screening and structural analysis, which identified SEC61A1 as one of the targets of mycolactone action [11, 13, 15, 40].” **(page 28, lines 480-482).**

2)	Introduction p. 5. Here, the authors state that the essential host factors that mediate the action of mycolactone remain largely unknown. In fact, both the Demangel and Simmonds laboratories have demonstrated that in mammalian cells, the direct target of mycolactone responsible for the compound cytotoxicity is the Alpha subunit of the Sec61 complex. The first papers in this are (PMID: 24699819, 27821549). It is required that this previous determination of the cellular target of mycolactone is at least mentioned, and the relevant literature is cited.

**Response:**

We thank the reviewer for this comment. According to the suggestion, we have discussed the previous reports about Sec61 as a mycolactone target and reported mediators of mycolactone-induced cell death (Hall *et al*; PMID: 24699819 and Baron *et al*; PMID: 27821549,) (references 7 and 11) in the **Introduction** section.

*“In vitro* studies showed that mycolactone blocks the translocation of nascent proteins across the ER membrane, and similar inhibitors such as CT7, CT8, and ipomoeassin F directly target the ER Sec61 translocon [7-12]. The SEC61α amino acid mutation R66G partly rescued mycolactone-induced cell death and protein translocation in HEK293 cells [11]. A structual study also showed that mycolactone binds the Sec61 translocon [13]. On the other hand, a haploid genetic screen revealed that the histone methyltransferase SETD1B is a mediator of mycolactone-induced cell death in KBM-7 cells, a chronic myelogenous leukemia cell line [14]. The activating transcription factor 4 (ATF4) also affects cell death pathways through the ER stress response in HeLa cells [15].” **(page 5, lines 93-104)**

3)	If I understand it correctly, the CRISPR screen carried out here is resulting in indels on the target gene, presumably resulting in abolished expression of the target gene product. This is of course problematic for identifying essential genes such as SEC61A1 and this can be circumvented by use of CRISPRi screens. It is not immediately clear to me from the text whether this as a CRISPR KO or a CRISPRi screen. This should be clarified and if a KO screen, then it should be discussed how the essential SEC61A1 came up as a hit.

**Response:**

Thank you for this important comment. We used the GeCKO v2 library as CRISPR knockout screening, not CRISPRi screen. This method is widely used for finding novel factors in toxicity pathways. Although CRISPR knockout screening is problematic for identifying essential genes compared with CRISPRi, SEC61A1 was identified as a top hit gene by genome screening in THP-1 cells. *SEC61A1* knockout THP-1 cells have low growth rates but can survive in the presence of mycolactone. We have added text in the **Introduction** section to clarify how we used the library.

“Therefore, we employed CRISPR knockout screening with human GeCKO sgRNA libraries to conduct comprehensive and unbiased loss-of-function screens to identify genes necessary for mycolactone-induced cell death in human premonocytic THP-1 cells.” **(page 6, lines 112-115)**

We confirmed Indel formation in the *SEC61A1* genome sequence in the revised manuscript **(S2 Fig C)**. Our results indicate that *SEC61A1* is not an essential gene for the survival of THP-1 cells. We have mentioned that *SEC61A1* knockout affects cell viability and growth rate in the **Discussion** section.

“SEC61A1 has an important role in protein translocation and calcium leakage [42, 46]. It was reported that *SEC61A1* deletion or silencing resulted in growth defects or cell death in association with sustained *XBP1* splicing [34, 35, 50]. In our study, deletion of *SEC61A1* showed a low growth phenotype and sustained *XBP1* splicing in THP-1 cells (Fig 5C and S2 Fig D). However, *SEC61A1* deletion did not induce apoptosis or the expression of proapoptotic genes such as *PMAIP1*, *BBC3* and *BCL2L11* (Fig 5B). We could not elucidate the mechanism contributing to the different growth and cytotoxic phenotype of *SEC61A1* deletion between THP-1 cells and other cells. In our genome-wide screening, *SEC61A1*-knockout cells had low growth rates but could survive in the presence of mycolactone; *SEC61A1* was found as a top score gene (Table 1).” **(page 29, lines 514-524).**

4)	Page. 19, Fig. 3. The followup screen clearly shows that cells transduced with sgRNAs against SEC61A1 are highly resistant against mycolactone cytotoxicity. Given the cell-essential nature of Sec61 in all eukaryotic cells, I am slightly confused about this result. Typically, full blockade or downregulation of Sec61 is highly toxic to cells already around 72 hours (see e.g. PMID: 32067014). The authors should discuss this unexpected result in the text. Perhaps the SEC61A1 KO was not complete in these experiments. Does the sgRNA targeting of SEC61A1 and the used mycolactone treatment block new synthesis of secreted and membrane proteins? This could be tested by for example WB analysis of known Sec61 substrate proteins expressed in THP-1 cells.

**Response:**

We thank the reviewer for this comment. The viability of the sgRNA treated THP-1 cells without mycolactone is 100% by the trypan blue exclusion assay. *SEC61A1*-knockout cells did not decline in our study. *SEC61A1*-knockout THP-1 cells were selected with 0.5 μg/mL puromycin for 1 week. The surviving cells were reseeded and expanded to a large enough number to experiment for 2 weeks. *SEC61A1*-knockout cells have low growth rates but can survive without declining in cell number. We have added a description of the puromycin selection of the sgRNA-treated cells in the **Methods** section.

“THP-1 cells were incubated with lentiviral vectors for 16 h in the presence of 8 μg/mL polybrene (Sigma-Aldrich) for lentivirus transduction, then selected with 0.5 μg/mL puromycin (Sigma-Aldrich) for at least 1 week. Surviving cells were reseeded in a 10 cm dish and expanded to use for the experiments.” **(page 10 lines 193-197)**.

We also discussed the cell viability and growth of *SEC61A1* knockout cells in the **Discussion** section.

“SEC61A1 has an important role in protein translocation and calcium leakage [42, 46]. It was reported that *SEC61A1* deletion or silencing resulted in growth defects or cell death in association with sustained *XBP1* splicing [34, 35, 50]. In our study, deletion of *SEC61A1* showed a low growth phenotype and sustained *XBP1* splicing in THP-1 cells (Fig 5C and S2 Fig D). However, *SEC61A1* deletion did not induce apoptosis or the expression of proapoptotic genes such as *PMAIP1*, *BBC3* and *BCL2L11* (Fig 5B). We could not elucidate the mechanism contributing to the different growth and cytotoxic phenotype of *SEC61A1* deletion between THP-1 cells and other cells. In our genome-wide screening, *SEC61A1*-knockout cells had low growth rates but could survive in the presence of mycolactone; *SEC61A1* was found as a top score gene (Table 1)." **(page 29, lines 514-524).**

5)	Page 25. “Therefore, we speculate that mycolactone directly binds to SEC61A1, thereby inhibiting modification of signaling peptides and calcium leakage, which results in accumulation of unfolded proteins in the ER. This process in turn causes ER stress”. This sentence needs to be rewritten. First, it is well established that mycolactone directly binds Sec61 (PMID: 27821549, 32692975) and this should be acknowledged in the text. Second, mycolactone inhibits protein translocation into the ER primarily by blocking access of polypeptides into the ER lumen (or ER membrane for integral membrane proteins). Signal peptide cleavage (not signalling peptide) occurs after the signal peptide has passed through the channel.

**Response:**

Thank you for these comments. According to the suggestion, we have discussed this previous work in the **Discussion** section and added the suggested references (Baron *et al*; PMID: 27821549, and Gerard *et al*; PMID: 32692975) (references 11 and 13).

“In addition, other inhibitors that directly target SEC61A1, such as CT7, CT8, and ipomoeassin F, have similar effects to mycolactone [8-12]. Mycolactone physically binds to SEC61A1, maintains the Sec61 translocon conformation, and prevents the access of signaling peptides to the binding site of SEC61A1 [13]. Mycolactone alters the Sec61 translocon conformation to open the cytosolic side of the lateral gate and enhance calcium leakage [44]. Mycolactone also prevents the translocation of proteins that pass through the endoplasmic reticulum for secretion or placement in cell membranes [7, 8, 45]; these proteins accumulate and activate ER stress responses [7, 46]. In the present study, we demonstrated that the knockout of *SEC61A1* blocked the mycolactone-induced expression of ER stress-related genes, eIF2α phosphorylation, and apoptosis in human premonocytic THP-1 cells. Therefore, mycolactone directly binds to SEC61A1 thereby inhibiting the modification of signaling peptides and enhancing calcium leakage, which results in the accumulation of mislocated proteins [8, 46, 47].” **(page 28, lines 497-509)**

Minor points:
1)	In the abstract, the authors state that SEC61A1 could be a therapeutic target for Buruli ulcer disease, but I am not convinced how Sec61 could be targeted for this purpose. The authors should clarify their rationale on how modulation of Sec61 activity could produce therapeutic benefits against Buruli ulcer disease keeping in mind that global blockade of Sec61 activity is generally cytotoxic to all eukaryotic cells.

Response:

Thank you for pointing out this important issue. We meant “inhibition of mycolactone activity”, but it was not clear in the text. To clarify the reviewer’s concern, we have modified the **Abstract** section.

“These results shed light on the cytotoxicity of mycolactone and suggest that the inhibition of mycolactone activity or SEC61A1 downstream cascades will be a novel therapeutic modality to eliminate the harmful effects of mycolactone in addition to the 8-week antibiotic regimen of rifampicin and clarithromycin.” **(page 3, lines 48-52)**.

2)	Page 13. The authors state that significant THP-1 cell death was induced by 30 ng/ml mycolactone at 48 hours. How does this compare to IC50 values reported elsewhere?

Response:

Thank you for this comment. It was reported that murine L929 fibroblast cells had shown an IC50 of 5 nM (3.715 ng/mL, mycolactone MW: 743) at 48 h with mycolactone as the lethal concentration (Scherr *et al.*; PMID: 23556027) (reference 33). Human embryonic lung fibroblast cells had shown 50% cytotoxicity at a concentration of 4.7 ng/mL (Sarfo *et al.*; PMID: 24731247) (reference 32). In this paper we have shown significant cell death with 30 ng/ml mycolactone at 48 h and did not show significant cell death with 3 ng/mL mycolactone in THP-1 cells. According to the suggestion, we have mentioned a comparison of mycolactone concentration in other papers.

“Significant cell death was induced by 30 ng/mL synthetic mycolactone at 48 h after treatment in THP-1 cells, as evidenced by the trypan blue exclusion assay (Fig 1A). This concentration is 6 to 8 times higher than the previously reported concentrations used in other cell types [32, 33].” **(page 14, lines 283-286)**.

3)	Page 15. Authors write that cells were mutagenized using lentiviral gRNA delivery. Is mutagenized the correct word here

**Response:**

Thank you for this comment. We have modified “mutagenized” to “genome-edited” as follows,

“Cells were genome-edited using the lentivirus-based human GeCKO v2 library consisting of 123,411 sgRNAs targeting 19,050 protein-coding genes and 1,000 control genes.” **(page 16, lines 320-322)**.

4)	Page 16. “Cells were then treated with mycolactone, and genomic DNA was prepared from the surviving cells”. Please state the concentration used and the duration of compound treatment.

**Response:**

We have mentioned the concentration and the duration of mycolactone treatment in the revised manuscript.

“Cells were then treated with 30 ng/ml mycolactone for 1 week, and the surviving cells were recovered and reseeded to use for genomic DNA sequencing.” **(page 17, line 324-326)**.

5)	Page 19. “We next explored the possibility that SEC61A1 is involved in mycolactone induced apoptosis.”. As mentioned, the role of Sec61 in mycolactone cytotoxicity is well established and this sentence should be reworded maybe to highlight the less studied form of apoptosis that mycolactone induces.

**Response:**

According to the suggestion, we have modified the text to emphasize that we explored the apoptosis pathways induced by mycolactone in THP-1 cells in this paper.

“We next explored the possibility that SEC61A1 is involved in mycolactone-induced apoptosis, especially focusing on the molecular mechanisms of apoptosis in THP-1 cells.” **(page 20, lines 374-376)**.

6)	Page 22. The authors discuss the role of mycolactone for inducing a ER stress response. Here, a citation to a proteomic study characterizing the specific form of proteotoxic stress induced by mycolactone was characterized (PMID: 29915147).

**Response:**

According to the suggestion, we have mentioned this previous proteomic study with the suggested reference (Morel *et al*; PMID: 29915147: reference 46) in the **Discussion** section.

“Morel *et al*. demonstrated that mycolactone induced an atypical ER stress response, in which the ATF4/DDIT3 branch of the ER stress response was robustly activated, but *Bip*, an upstream regulator of the ER stress response, was downregulated [39, 46]. It was different from the conventional ER stress response induced by thapsigargin, tunicamycin, and MG132. Our study also showed mycolactone-induced *ATF4/DDIT3* expression, which was diminished by *SEC61A1* knockout. *ATF6* gene activation was induced only by thapsigargin and not by mycolactone. We have confirmed that the SEC61A1 knockout suppressed mycolactone-induced ER stress and cytotoxicity.” **(page 31, lines 560-568)**.

7)	Page 24. Sec61 protein complex is typically written as Sec61, not SEC61.

**Response:**

Thank you for this comment. According to the suggestion, we have modified SEC61 protein complex to Sec61 protein complex throughout the revised manuscript.

8)	Page 26. Again, please clarify how Sec61 modulation could allow therapeutic targeting of Buruli ulcer disease.

**Response:**

As we had discussed in our response to minor point #1, it would be beneficial to block the toxic effects of mycolactone to modulate SEC61A1 and downstream signaling pathways for therapeutic purposes. However, there are no effective drugs to modulate this pathway. So, we have mentioned other approaches to reduce the toxicity of mycolactone and further studies for the purpose of treatment in the **Discussion** section.

“Several approaches have been proposed to inhibit mycolactone activity, *e.g.,* the use of IgG antibody against mycolactone [57, 58], the inhibition of the mycolactone synthesis pathway with substrates (malonyl-CoA, methyl malonyl-CoA) [59] and the attenuation of cytotoxicity with antioxidants [48, 49]. At present there is no effective drug to reduce the toxicity of mycolactone, and further drug screening and analysis of the biological activity of mycolactone will be required to develop novel therapeutic modalities to inhibit mycolactone-induced ulceration.” **(page 32, lines 573-579)**.
