# Supplementary material for: Genome-wide screening identified SEC61A1 as an essential factor for mycolactone-dependent apoptosis in human premonocytic THP-1 cells
Source: PLoS Negl Trop Dis. 2022 Aug 8;16(8):e0010672. doi: 10.1371/journal.pntd.0010672 (PMC9387930; doi:10.1371/journal.pntd.0010672)
Supplement: Attachment — Submitted filename: Suzuki K-Mycolactone response220704AK.docx [file pntd.0010672.s009.docx]

**Methods**
Reviewer #2: (No Response)
Reviewer #3: The methods for addressing the questions raised by the reviewers are appropriate.

**Response:**

We appreciate your positive comments.

--------------------
**Results**
Reviewer #2: (No Response)
Reviewer #3: The results are sufficiently presented with the exception of the Western blot images in Figures 1,4 and 5. It is difficult to assess the quality of the Western blotting data as the images are cropped very close to the bands. I would recommend including uncropped WB images in the Supplementary Data section.

**Response:**

According to the suggestion, we have added original uncropped images of Western blots in S4 Fig. And we have mentioned S4 Fig in the **Materials and Methods** section**.**

“*Original images of Western blots are shown in S4 Fig.*” **(Page 12, lines 241)**

--------------------
**Conclusions**
Reviewer #2: (No Response)
Reviewer #3: Conclusions are sufficiently supported by the data and study limitations are also discussed suitably.

**Response:**

We thank the reviewer for this comment.
--------------------

**Editorial and Data Presentation Modifications?**
Reviewer #2: Minor Points

1. Line 447. "Thasigargin also induced IRE1alpha and EIF2alpha phosphorylation". This sentence is ambiguous as it could be interpreted as indicating phosphorylation of IRE1 rather than an increased protein expression. Please rewrite making the meaning clear.

**Response:**

We have amended the sentence as suggested.

“*Thapsigargin also induced IRE1α protein synthesis and eIF2α phosphorylation in all these cells.*” **(Page 25, lines 447-448)**

2. Line 491 . " The Sec61 complex also functions as a channel that mediates calcium leakage from the ER, a process required for stabilization if protein folding". This makes it sound as if it is the calcium leakage that stabilises folding whereas it is the calcium within the ER which is required for protein folding and the loss due to leakage will lead to destabilisation. Please rewrite this sentence to make this clear.

**Response:**

We have amended the sentence as suggested.

“*The Sec61 complex also functions as an ER Ca^2+^ channel that modulates calcium homeostasis. Ca^2+^ is required for protein folding in the ER and its leakage will lead to destabilization of proteins [43].*” **(Page 28, lines 491-493)**

3. Line 508. "..thereby inhibiting the modification of signalling peptides.." It is not the inhibition of signal peptide modification which is causing the mislocalization but the inhibition of translocation into the ER. This sentence needs to be corrected.

**Response:**

We have amended the sentence as suggested.

“*Therefore, mycolactone directly binds to SEC61A1 thereby inhibiting the protein translocation into the ER and enhancing calcium leakage, which results in the accumulation of mislocated proteins [8, 46, 47].*” **(Page 29, lines 507-509)**

4. Line 569. "... SEC61A1 knockout was not completely prevented by mycolactone-induced apoptosis.". I assume you mean mycolactone-induced aopotosis was not completely blocked by SEC61A1 knockout.

**Response:**

We have amended the sentence as suggested.

“*However, the viability assay and FLICA assay demonstrated that mycolactone-induced apoptosis was not completely prevented by SEC61A1 knockout.*” **(Page 32, lines 568-569)**

5. Line 587. "... to block mycolactone by inhibiting SEC61A..." Inhibition of SEC61A1 will NOT block the actions of mycolactone. Please take this out or rephrase to make it clear that you mean targeting of downstream effects.
**Response:**

Thank you for pointing out important issue. “Block mycolactone by inhibiting SEC61A1” was an error in wording. This sentence had been deleted**.**

Reviewer #3: I would recommend accepting the article in the current form if full Western blotting images are provided.

**Response:**

Thank you for the comment. We have added original uncropped images of Western blots from in S4 Fig.

--------------------
Reviewer #2: The authors have adequately addressed most of my concerns. I think the paper should be accepted if the above editorial amendments are made

Reviewer #3: The authors have addressed all of the reviewer suggestions by new experiments or modifications to the text. I recommend publication of the article in the current form.

**Response:**

We thank the reviewers for the kind comments and valuable feedback.
